# High-Efficiency Wavelet Compressive Fusion for Improving MEMS Array Performance

**DOI:** 10.3390/s20061662

**Published:** 2020-03-17

**Authors:** Siyuan Liang, Weilong Zhu, Feng Zhao, Congyi Wang

**Affiliations:** Key Laboratory of Information Communication Network and Security, Xi’an University of Posts and Telecommunications, Xi’an 710121, China; telestorm@163.com (S.L.); hfengzhao@xupt.edu.cn (F.Z.); congyiwang@stu.xupt.edu.cn (C.W.)

**Keywords:** inertial navigation, MEMS array, compressive fusion

## Abstract

With the rapid development of microelectromechanical systems (MEMS) technology, low-cost MEMS inertial devices have been widely used for inertial navigation. However, their application range is greatly limited in some fields with high precision requirements because of their low precision and high noise. In this paper, to improve the performance of MEMS inertial devices, we propose a highly efficient optimal estimation algorithm for MEMS arrays based on wavelet compressive fusion (*WCF*). First, the algorithm uses the compression property of the multiscale wavelet transform to compress the original signal, fusing the compressive data based on the support. Second, threshold processing is performed on the fused wavelet coefficients. The simulation result demonstrates that the proposed algorithm performs well on the output of the inertial sensor array. Then, a ten-gyro array system is designed for collecting practical data, and the frequency of the embedded processor in our verification environment is 800 MHz. The experimental results show that, under the normal working conditions of the MEMS array system, the 100 ms input array data require an approximately 75 ms processing delay when employing the *WCF* algorithm to support real-time processing. Additionally, the zero-bias instability, angle random walk, and rate slope of the gyroscope are improved by 8.0, 8.0, and 9.5 dB, respectively, as compared with the original device. The experimental results demonstrate that the *WCF* algorithm has outstanding real-time performance and can effectively improve the accuracy of low-cost MEMS inertial devices.

## 1. Introduction

Microelectromechanical systems (MEMS) devices have many advantages in terms of size, power consumption, reliability, and impact resistance [1], and they have been widely used in the medical, industrial, and transportation fields, as well as other civil fields. MEMS inertial sensors are very important MEMS devices. To further expand the application range of MEMS gyroscopes, the development of methods for improving the measurement accuracy of MEMS gyroscopes using existing technology is a popular topic in the field of inertial navigation.

An important method for improving sensor output accuracy is to use multisensor data fusion technology. A MEMS array [2] measures the angular velocity of a given carrier with multiple MEMS gyroscopes and, then, uses data fusion to obtain the optimal estimate. This technique is also referred to as ”virtual gyroscope” technology, and it was first proposed by the Jet Propulsion Laboratory (JPL) in the United States. In 2003, the JPL personnel simulated four gyroscopes with drifts of 8.66°/h. Under static conditions, when the correlation coefficient between gyros is −0.333, the drift of the gyro is reduced to 0.062°/h [3]. Xue Liang et al. [4] studied the correlation of a MEMS array and demonstrated its influence on the angular velocity accuracy signal. Previous studies [5,6] presented a design method and a fusion estimation method using MEMS arrays under different scenarios. Moschas Fanis et al. [7] proposed a simple and effective method for testing the performance of state-of-the-art MEMS arrays in various engineering applications. In addition, with the continuous development of wavelet technology [8], the superior performance of wavelets in filtering has led to extensive applications in the field of signal processing.

Currently, combining virtual gyroscope technology with other filtering technologies is becoming increasingly prevalent. Martin Tanenhaus et al. [9] combined denoised sensor data with the Kalman filter offset compensation algorithm to lower the bias stability (standard deviation) of the gyro to less than 0.1 degrees/hour. Kuan-Ying Huang et al. [10] combined a MEMS array with M-estimation filters to effectively suppress non-Gaussian impulse noise and provide angular velocity measurements with high accuracy. Wavelet filtering does not suppress non-Gaussian impulse noise well. Because virtual gyroscope technology provides good suppression of non-Gaussian pulse signals, this paper proposes a wavelet compressive fusion (*WCF*) algorithm for MEMS arrays based on wavelet filtering [11] and multisensor data fusion [12]. In general, the combination of MEMS data fusion technology and other filtering technologies should considerably improve sensor accuracy; however, large processing delays could result, which is a disadvantage. Thus, improving the accuracy intractably contradicts the decrease in the processing delay.

In this paper, we contribute to the literature by proposing compressive fusion and a realizable compressive fusion method based on wavelet theory and data fusion technology. The experimental results show that the proposed *WCF* algorithm effectively suppresses additive white Gaussian noise and non-Gaussian impulse noise but also greatly reduces the processing delay. Section 2 describes the algorithm in detail. Section 3 discusses the simulation test and result analysis. Section 4 presents the practical experiment and result analysis, focusing on the configuration of the experimental hardware platform, experimental results, and analysis. Section 5 presents the conclusions.

## 2. Materials and Methods

### 2.1. Random Error Model of a MEMS Array

On the basis of the random error model of a MEMS gyroscope, we can obtain the random error model [13] of the MEMS gyroscope array, which is shown in Equation (1).

(1) Y=I·d+B+n

(2) $$\dot{B}={\left[{\bar{w}}_{1}\;{\bar{w}}_{2}\cdots {\bar{w}}_{N}\right]}^{T}$$

Here, $Y={\left[Y_{1}\;Y_{2}\cdots Y_{N}\right]}^{T}$ is the rate vector of the gyroscope output angle; $B={\left[B_{1}\;B_{2}\cdots B_{N}\right]}^{T}$ represents the vector of angular rate random walk, which can be represented as noise driven by random walk vector $\dot{B}$; n is the vector of angular random walk; N is the number of gyroscopes in the array; d represents the real angular rate vector of the MEMS gyroscope array; and $I={\left[1\;1\cdots 1\right]}^{T}$.

### 2.2. Compressive Fusion 

Signal compression can reserve the most significant characteristics of the original signal. Therefore, we assume that if we first compress the single sensor data, and then fuse the compressed data, we could obtain more precise data than the original signal data. This method can be expressed as shown below.

(3) $$D=\left[\begin{array}{ccccc} d_{1,1} & d_{1,2} & \cdots & d_{1,l-1} & d_{1,l}\\ d_{2,1} & d_{2,2} & \cdots & d_{2,l-1} & d_{2,l}\\ \vdots & \vdots & \vdots & \vdots & \vdots \\ d_{N-1,1} & d_{N-1,2} & \cdots & d_{N-1,l-1} & d_{N-1,l}\\ d_{N,1} & d_{N,2} & \cdots & d_{N,l-1} & d_{N,l} \end{array}\right]$$

We can use $D_{i,l}$ to represent $\left[d_{i,1}\;d_{i,2}\cdots d_{i,l}\right]$, which indicates that the data length of the i-th gyroscope is l. Thus,

(4) $$D={\left[D_{1,l}\;D_{2,l}\cdots D_{N,l}\right]}^{T}$$

We use fusion technology to compress D, and Compression ( ) is used to represent any type of compression method. This representation is conceptual, and different compression methods may perform differently.

(5) $$D^{\prime }={\left[D_{1,l^{\prime }}\;D_{2,l^{\prime }}\cdots D_{N,l^{\prime }}\right]}^{T}=Compression(D)$$

$D^{\prime }$ is the compressed signal. When $l^{\prime }\leq l/2$, there is a good compression effect. Under the premise of being able to accurately recover the compressed signal, the smaller the value of l, the better.

The fusion equation based on compressed array data, which is easy to obtain, is shown in Equation (6):

(6) $$\mathrm{EVA}=D^{\prime }\cdot \left[\begin{array}{c} \begin{array}{c} \mu_{1}\zeta_{1}\\ \mu_{2}\zeta_{2} \end{array}\\ \vdots \\ \mu_{N-1}\zeta_{N-1}\\ \mu_{N}\zeta_{N} \end{array}\right]$$

where $\mu_{i}$ is the weight of the i-th sensor, and $\xi_{i}$ is the correlation coefficient. This equation is a general data fusion equation, and it represents the general form of array data fusion.

### 2.3. Design of Wavelet Compressive Fusion

In this paper, we apply wavelet technology to compress the signal. The Mallat algorithm [14] is a fast wavelet transform algorithm that greatly increases the operation speed of wavelet multiscale analysis and signal reconstruction. The wavelet transform output of the i-th gyroscope is $x_{i}=\left[c_{i,N}\;d_{i,N}\cdots d_{i,1}\right]$. $c_{i,j+1}$ and $d_{i,j+1}$ represent the compressed signal data and detailed wavelet coefficient of the i-th gyroscope at the j+1 layer after the transform.

This paper adopts a support degree-based fusion algorithm. According to the characteristics of the gyroscope output data, the support function shown in Equation (6) is constructed in the algorithm:

(7) $$r_{ij}=\exp\left\{\frac{-\psi \left[c_{i,N}(k)-c_{j,N}(k)\right]}{\bar{c}}\right\}$$

where ψ represents the sensitivity of the support degree to the difference between measured values, and $\bar{c}$ represents the correlation coefficient of the sensor array. $c_{i,N}$ represents the compressed signal data of the i-th gyroscope after the N-layer wavelet transform. $r_{ij}$ represents the support degree of the j-th gyroscope to the i-th gyroscope (1≤i,j≤n, where n is the number of gyroscopes). The compressed signal data are used to calculate the support degree between different gyroscopes, and then the weight of each gyroscope is obtained. For different sensor array structures, the value of ψc¯ will be different, which will affect the performance of the proposed algorithm to some degree. The support function used in this paper performs better than existing support functions for addressing MEMS gyroscope data.

The comprehensive support function is

(8) $$s_{i}=\sum_{j=1,j\neq i}^{n}r_{ij},\;i=1,2,\cdots ,n$$

where $s_{i}$ indicates how the decisions of other gyroscopes depend on the i-th gyroscope.

The consistency measurement function is

(9) $$\xi_{i}(k)=\frac{s_{i}}{n-1},\;i=1,2,\cdots ,n\;0<\xi_{i}(k)\leq 1$$

Reference [15] considers the mean value $\bar{\xi_{i}}(k)$ and the variance value $\sigma_{i}^{2}(k)$ of the consistency value at moment k of the i-th sensor. The weight assignments of different sensors derived from the mean and variance of the consistency values are

(10) $$\mu_{i}(k)=\frac{\bar{\xi_{i}}(k)}{0.1+\lambda \sigma_{i}^{2}(k)},\;i=1,2,\cdots ,n$$

Data fusion estimation is performed as follows:

(11) $$c_{N}(k)=\frac{\sum_{i=1}^{N}\left[\mu_{i}(k)c_{i,N}(k)\right]}{\sum_{i=1}^{N}\mu_{i}(k)},\;i=1,2,\cdots ,n$$

In Equation (10), $c_{i,N}$ is the compressed signal data of the i-th sensor after N layers of the iterative wavelet transform, and $c_{N}$ represents the data fusion estimate of the MEMS array compressed signal data after the iterative wavelet transform.

### 2.4. Detailed Coefficient Selection and Signal Reconstruction

The average of the detailed wavelet coefficients is used to estimate the detailed wavelet coefficients. The optimal estimate of the wavelet coefficient is

(12) $$x=\left[c_{N}\;\bar{d_{N}}\cdots \bar{d_{1}}\right]$$

Threshold processing largely determines the final effect of wavelet processing; thus, the choice of threshold is very important. The threshold calculation equation used in the algorithm is

(13) $$T_{i}=\sigma_{i}\sqrt{2\cdot \ln N}\;i=1,2,\cdots ,n$$

where $\sigma_{i}$ is the noise coefficient of the i-th sensor, and $T_{i}$ is the global threshold of the i-th sensor.

The global threshold fusion is

(14) $$T=Fusion\_function([T_{1},T_{2},\cdots ,T_{n}])$$

where Fusion_function () is the process of data fusion based on the support degree.

To maintain the continuity of the signal, the soft threshold processing method is adopted. The equation is as follows:

(15) $$x^{\prime }(k)=\{\begin{array}{cc} \mathrm{sign}(x(k))(\left|x(k)\right|-T), & \left|x(k)\right|>T\\ 0, & \left|x(k)\right|\leq T \end{array}$$

During signal reconstruction, the original address operation is used in the wavelet transform and wavelet inverse transform, which reduces the resource overhead of the operation and also improves system efficiency.

The implementation process of the *WCF*-based MEMS array optimal estimation algorithm designed in this paper is shown in Algorithm 1.
**Algorithm 1** WCF Algorithm 1:**INITIALIZATION:** 2:Sensor number: i=1; Number of sensors: n; Number of decomposition levels: N. 3:**read** Ten sets of measured data ($z_{1}∼z_{10}$). 4:**load** Filter coefficient ($g_{d},h_{d}$). 5:**WAVELET DECOMPOSITION:** 6:**repeat** 7:Perform multiscale wavelet compression on $z_{i}$. 8:Obtain compression signal data $c_{i,N}$ and detailed part $d_{i}=\left[d_{i,N}\cdots d_{i,1}\right]$ of the wavelet composition. 9:i←i+1. 10:**until**i=n. 11:**FUSION ESTIMATION:** 12:Conduct data fusion on $c_{1,N}∼c_{n,N}$ and obtain the fusion estimate $c_{N}$. 13:Obtain the average d of $d_{1}∼d_{n}$. 14:Obtain optimal estimation $x=\left[c_{N}\;\bar{d_{N}}\cdots \bar{d_{1}}\right]$ of wavelet coefficients after fusion estimation. 15:Construct a threshold function and obtain the fused global threshold T. Then, perform threshold processing on x; the result is $x^{\prime }$. 16:**SIGNAL RECONSTRUCTION:** 17:**repeat** 18: Take the inverse wavelet transform. 19: Update the wavelet coefficients. 20: N←N−1. 21:**until**N=0. 22:**END**

### 2.5. Flowchart of the WCF Algorithm

**Figure 1:**
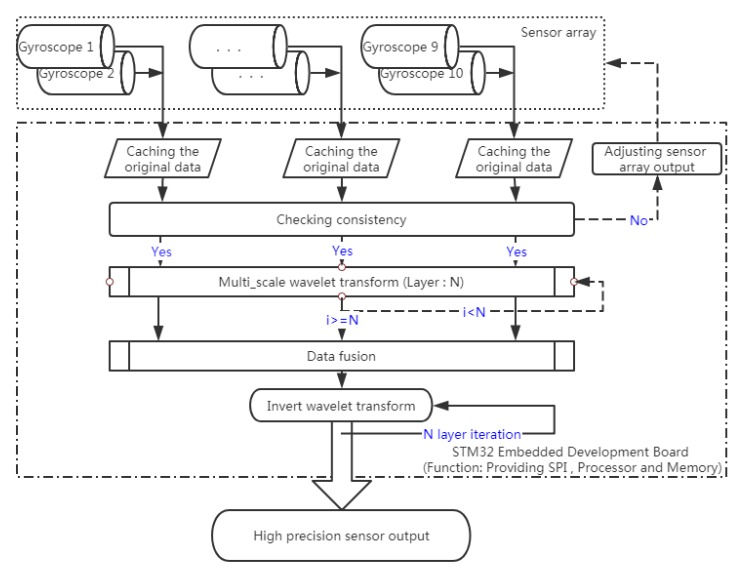
The flowchart of the *WCF* algorithm.

To further describe the *WCF* algorithm, we designed a flowchart for it, as shown in Figure 1.

## 3. Experiment and Results Analysis Based on Simulation Data

To verify the performance of the *WCF* algorithm, a simulation verification environment was set up on *MATLAB.* In our simulation test, db3 was chosen as the wavelet basis. To determine the decomposition level, we designed a group of auxiliary experiments by using different decomposition levels to test the simulated data. Auxiliary experiments showed that the suppression effect on noise was not obvious when the number of wavelet decomposition levels was less than five. The suppression effect on the noise improved when the number of layers was greater than five and less than eight, but the improvement was negligible when the computational complexity also increased. When the number of layers was greater than eight, information was lost, and the distortion became increasingly serious as the number of wavelet decomposition levels increased. Therefore, the decomposition level is set to five in the subsequent analysis.

### 3.1. Simulation Verification Environment

**Figure 2:**
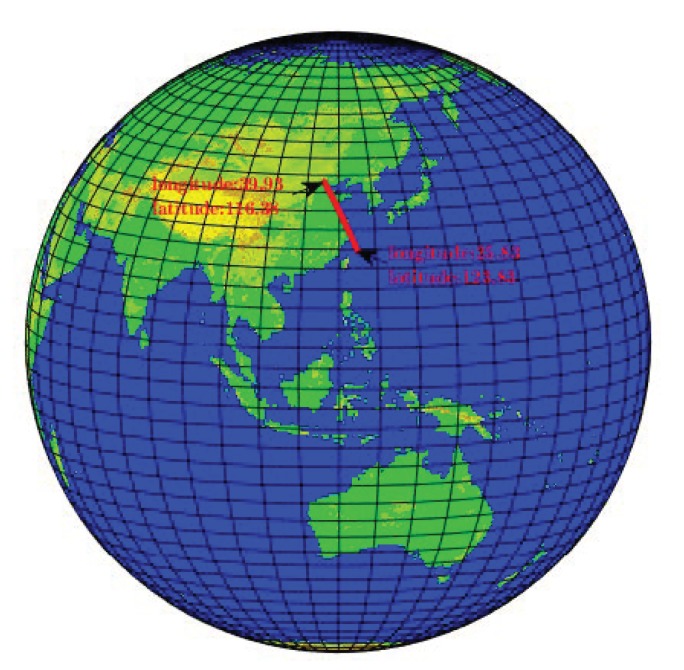
Ideal great circle trajectory.

A great circle path with a maximum height of 10 km was simulated, starting from a point in Beijing (longitude 39.93, latitude 116.38) and ending at a small island (longitude 25.83, latitude 123.83). The distance as the crow flies is approximately 1730 km, and the flight path of the aircraft is a great circle, with a maximum altitude of 10 km and a maximum flight speed of 500 nautical miles per hour. The trajectory top view is shown in Figure 2.

**Figure 3:**
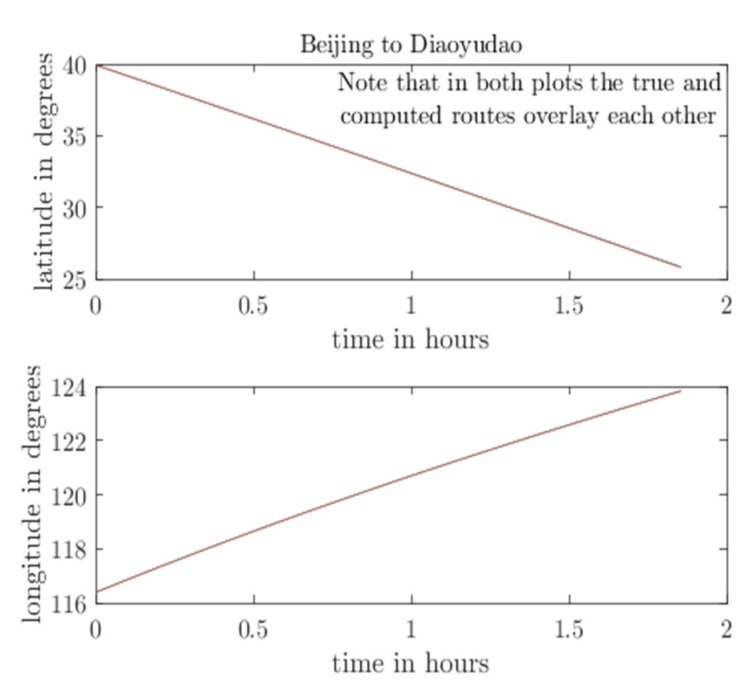
Coordinate variation during operation.

**Figure 4:**
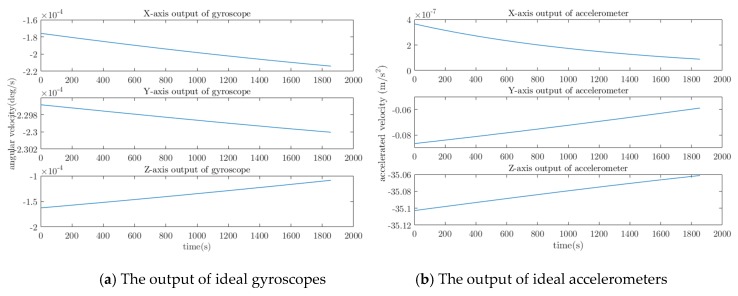
The output of ideal inertial sensor.

According to the trajectory, the flight path of the aircraft is simulated, and the coordinate changes in the flight path and the ideal sensor output are calculated in real time. The coordinate variation is shown in Figure 3, and the ideal sensor output is shown in Figure 4.

### 3.2. Error Introduction and Algorithm Optimization

**Figure 5:**
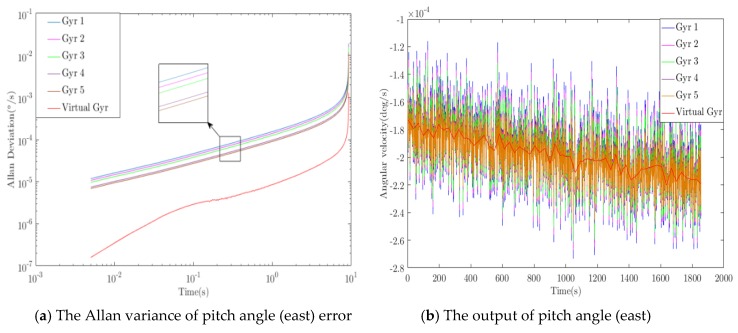
Optimization results of pitch angle (east) error.

**Figure 6:**
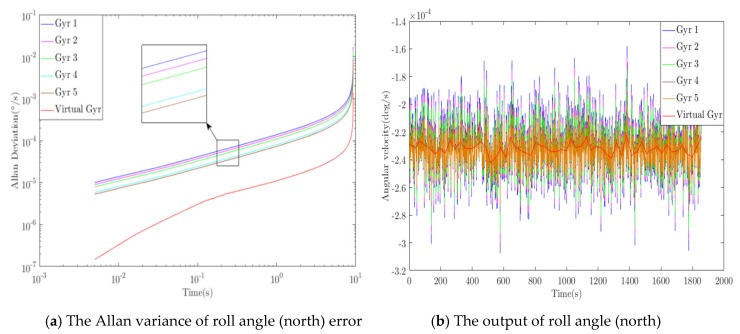
Optimization results of roll angle (north) error.

**Figure 7:**
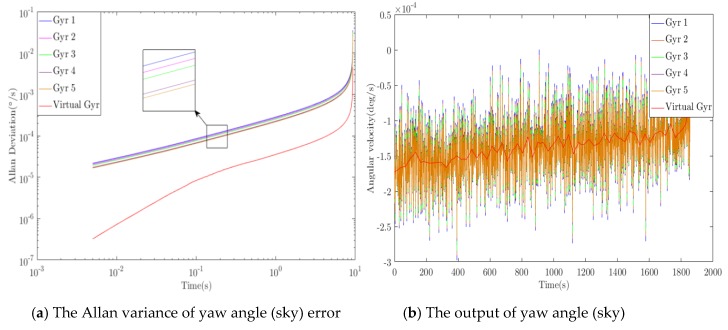
Optimization results of yaw angle (sky) error.

According to the ideal great circle trajectory, sensor error is introduced into the ideal sensor output, and the proposed algorithm is used to optimize the simulated output in real time during the flight of the aircraft. Figure 5, Figure 6 and Figure 7 show the optimization results of pitch angle, roll angle, and yaw angle. The quantitative analysis of the error is shown in Table 1, Table 2 and Table 3. If we were to show ten groups of gyroscope data in the figures, the figures would be too complicated. Thus, we show only five groups of the original gyroscope data in the above mentioned figures. The proposed WCF algorithm mainly improves the output performance of virtual gyroscope by using wavelet denoising and information fusion. The noise that affects the MEMS performance mainly includes Gaussian and non-Gaussian, and the wavelet denoising has good denoising effect on these two kinds of noise [16,17]. Redundant information is used for information fusion to improve the output performance and stability of the virtual gyroscope. The support matrix is adopted as the fusion method, which does not require too much prior information and has a high fusion accuracy [18,19].

### 3.3. Performance Evaluation of the WCF Algorithm for the Simulation

Analysis of the simulation result demonstrates that the proposed algorithm has optimal performance for pitching angle (east) error, roll angle (north) error, and yaw angle (sky) error. For the pitching angle (east) error, the *WCF* algorithm reduced the zero-bias stability and angular random walk to 1/9.5 and 1/8.9 of the best original data, and the ramp rate was reduced to 1/8.7 of the original. Using “dB’” to represent quantity relationship, those errors were improved by 9.8, 9.5, and 9.3 dB, respectively. For the roll angle (north) error, the errors were improved by 8.9, 8.2, and 8.2 dB, respectively. For the yaw angle (sky) error, the errors were improved by 7.6, 7.8, and 7.7 dB, respectively. During inertial navigation, the yaw angle error is generally the largest. Thus, compared with the pitching angle error and roll angle error algorithm, the optimization degree is slightly worse, which is consistent with the actual situation. Therefore, the simulation results demonstrate that the algorithm can effectively improve the output accuracy of the inertial sensor.

## 4. Experiment and Results Analysis Based on Sensor Measurement Data

Analysis of the simulation result demonstrates that the proposed algorithm has optimal performance for the output of the MEMS array. To further verify the performance of the *WCF* algorithm on the practical MEMS sensor array, we designed a practical verification environment for data acquisition and algorithm validation.

### 4.1. Verification Environment

The XV7011BB MEMS gyroscope sensor is used in the experimental system. This product is a single-axis digital gyroscope sensor manufactured by EPSON, and it has excellent zero-bias stability, low power consumption, and flexible modes. Specific performance parameters are shown in Table 4.

According to the literature [20,21], there are two design methods for MEMS gyro arrays. The first one manufactures several MEMS gyroscopes on the same chip, which is beyond the scope of this paper. The second one, which is used in this paper, fabricates several MEMS gyroscopes on a printed circuit board (PCB). A ten-gyro array system for collecting data was designed. Ten MEMS gyro sensors of the same batch and same model (XV7011BB) were selected for the experiment. The system was fixed on an experimental platform for experimental testing. The MEMS gyroscope has a data output frequency of 200 Hz.

**Figure 8:**
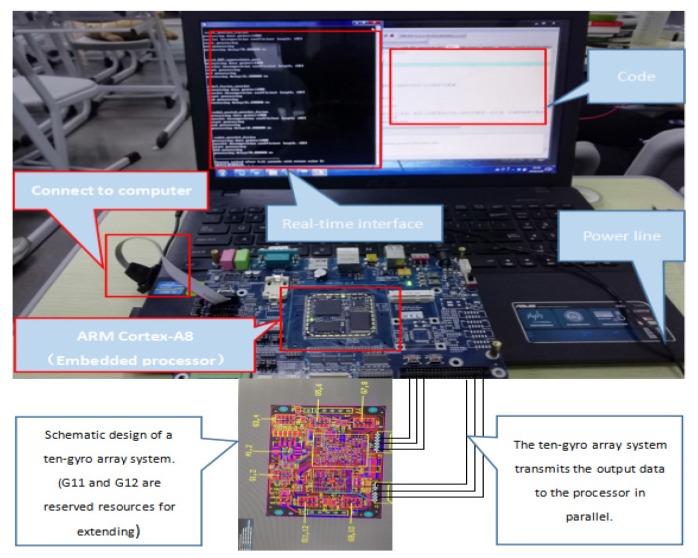
Hardware platform.

To evaluate the *WCF* algorithm, a hardware platform was established, as shown in Figure 8. The processor used in this environment was an ARM Cortex-A8 which runs at a speed of 800 MHz.

### 4.2. Online Data Processing

The measured data were processed according to the algorithm. First, we still used db3 as the wavelet basis. Second, the FIFO (first input, first output) stack was set to 1000. Every second, 20 data points were added to the stack, and then the data in the stack were processed. Obviously, this system is a memory system. Depending on the type of gyroscope, we can adjust the depth of the stack to satisfy its real-time requirements.

### 4.3. Performance Evaluation

**Figure 9:**
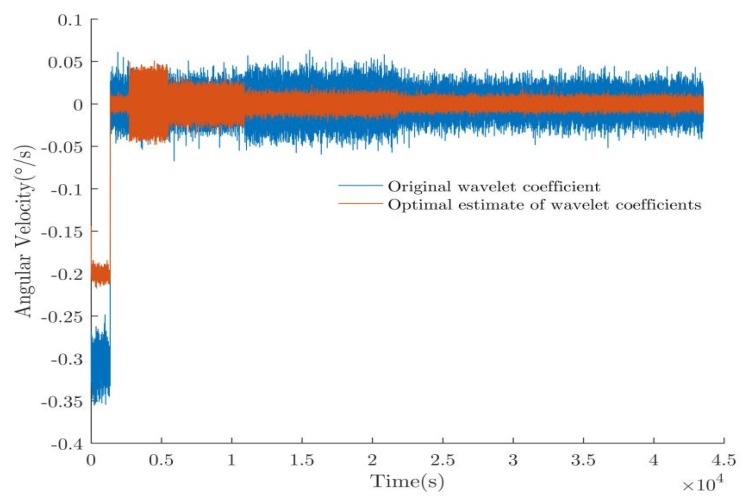
Comparison of the original wavelet coefficients and the optimal estimate of the wavelet coefficients.

A comparison of the wavelet transform of the original signal and the fusion result of the compressed signal data is shown in Figure 9. The divergence of the compressed signal is well suppressed after fusion processing, and the detailed wavelet coefficients clearly decreased after averaging.

**Figure 10:**
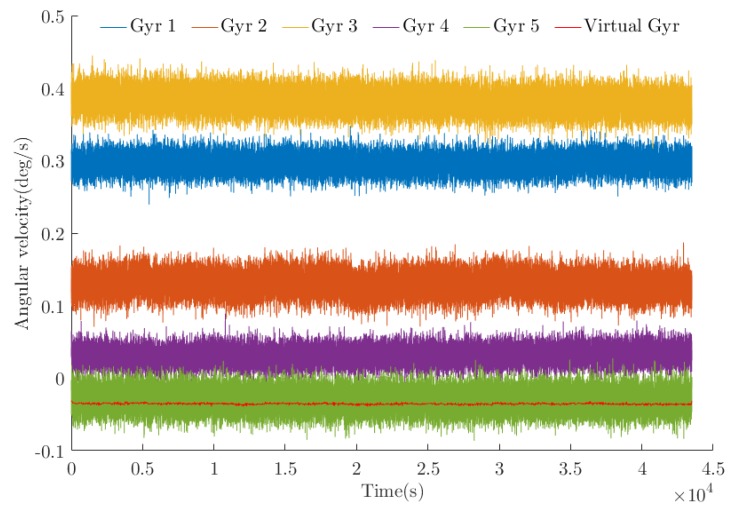
Processing results (only five groups of the original data are visualized).

**Figure 11:**
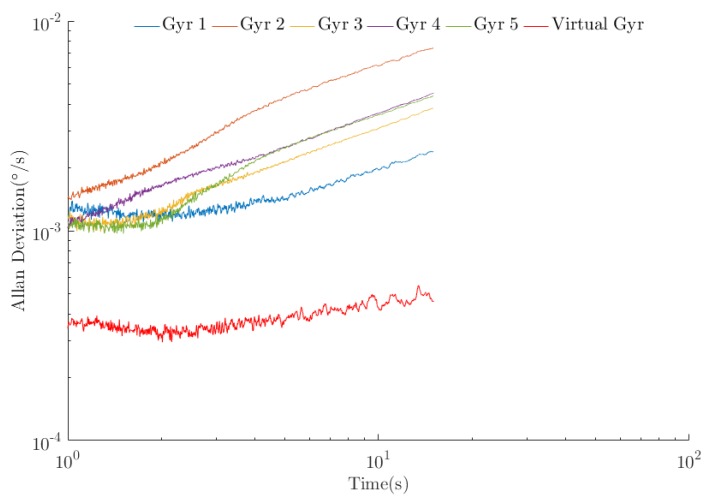
Double logarithmic graph of the Allan variance method (the Allan variances of the five original data points are visualized).

To observe the difference between the processed data and the original data more intuitively, five gyroscope data points in the sensor array were selected for visualization. A schematic diagram of the original signal and the optimal estimated value after the algorithm processing is shown in Figure 10. The angle random walk noise (ARW), zero-bias instability noise (BI), and rate ramp noise (RR) are important parameters for measuring gyroscope performance [22]. The Allan variance method [23,24] is currently the most effective method for calibrating the error of MEMS gyroscopes. In the experiment, the regression analysis method [25] was adopted to perform the Allan variance analysis for the original signal and the optimal estimated value. The bilogarithmic diagram is shown in Figure 11. The result produced by the *WCF* algorithm is the curve corresponding to the “virtual gyroscope”. A comparison to the original data shows that the trends of the curves are basically the same, indicating that data processing does not change the error characteristics of XV7011BB. The magnitude of the error was greatly reduced relative to the error amplitude of the original data, indicating that the various errors of the gyroscope were significantly weakened and that the degree of error improvement was basically the same and relatively balanced.

To illustrate the effect of the *WCF* algorithm, a control experiment based on a traditional algorithm was designed. The control group used independent wavelet filtering technology and independent support-based data fusion technology. Using this method, the selected data were processed online, and then an Allan variance analysis was performed. Additionally, the processing delays of the two methods were measured. The test method for the processing delay was to repeat each algorithm 10 times; the processing delay of each operation was recorded, and the statistical averages of the processing delays of the corresponding algorithms were calculated. The results of the *WCF* algorithm were compared with the results of the traditional algorithm, and the comparison results are presented in Table 5.

As Table 5 indicates, the processing results of the two algorithms significantly improved as compared with the precision of the device used. The traditional algorithm of “wavelet filtering + data fusion based on support degree” reduced the zero-bias stability, angle random walk, and ramp rate to 1/2.0, 1/3.1, and 1/1.7 of the best original data, respectively. The *WCF* algorithm reduced the zero-bias stability and angular random walk to 1/6.2 and 1/6.3 of the original values, and the ramp rate was reduced to 1/9.1 of the original. Using “dB” to represent quantity relationship, the errors were improved by 8.0, 8.0, and 9.5 dB, respectively. As the experimental data show, the proposed *WCF* algorithm can effectively suppress the main errors of the MEMS gyroscope. Additionally, the *WCF* algorithm suppresses the zero-bias stability, angular random walk, and ramp rate 3.1, 2.0, and 5.4 times more than the traditional algorithm, demonstrating its superiority. When processing 0.1 s of input array data, the *WCF* algorithm has an average processing delay of 0.075 s, whereas that of the traditional algorithm is 0.379 s. The proposed algorithm decreased the average processing delay by approximately 4/5 as compared with the traditional algorithm.

## 5. Discussion

In this paper, we focus on low-cost MEMS gyroscopes, which are one type of MEMS inertial sensor. In fact, the *WCF* algorithm can be applied to any type of MEMS inertial sensor because MEMS inertial sensors have almost the same error form [26]. Furthermore, the experimental results show that the proposed *WCF* algorithm can effectively suppress additive white Gaussian noise and non-Gaussian impulse noise. Therefore, we claim that the *WCF* algorithm could be applied to non-inertial sensors. We need to perform more experiments to verify this claim in future work.

Unfortunately, our verification test was performed under static conditions, and we did not test the performance of our algorithm under dynamic conditions due to the limitations of our lab equipment. According to conventional wisdom, the performance under dynamic conditions is reduced relative to that under static conditions, but not too significantly. Therefore, the *WCF* algorithm is not limited to static conditions, and we conclude that this method can be applied under dynamic conditions. In future work, we plan to further test the feasibility and performance of the *WCF* algorithm under dynamic conditions.

## 6. Conclusions

This paper presents a study on an optimal estimation method for MEMS gyro arrays. First, the problems and improvement directions of the current algorithm design were presented. Then, an optimal estimation algorithm for MEMS arrays based on *WCF* was proposed. The simulation result demonstrates that the proposed algorithm reaches the expected optimal performance on the output of the MEMS array. To further verify the performance of the *WCF* algorithm on the practical MEMS sensor array, a hardware platform based on the *WCF* algorithm model was built for experimental verification. This paper focuses on the precision improvement of the algorithm relative to the original data under static conditions. Then, the performance of the *WCF* algorithm was compared with that of the traditional algorithm. An Allan variance analysis was applied to calibrate the error of the output data and calculate the average processing delays of both algorithms. The results show that the *WCF*-based MEMS array optimal estimation algorithm can effectively improve the accuracy of the MEMS gyroscope. The experimental results indicate that under the normal working conditions of the MEMS array system, 100 ms of input array data (200 data) requires a processing delay of approximately 75 ms when applying the *WCF* algorithm, which is approximately four-fifths shorter than that of the traditional algorithm. Thus, the *WCF* algorithm can support real-time data processing of any inertial sensor, but the traditional algorithm cannot. In future work, we will test our algorithm by using dynamic cars to evaluate its dynamic performance. Meanwhile, we will verify the feasibility of compressive fusion in other engineering fields.

## Figures and Tables

**Table 1:** Optimization results of pitch angle (east) error.

Algorithm		Zero-Bias Stability °/*h*	Angle Random Walk °/*sqrt*(*h*)	Ramp Rate °/*h*^2^
Original signal	MAX	0.066	21.448	5.554
	MIN	0.038	12.495	3.236
WCF		0.004	1.407	0.371
Improved		9.8 dB	9.5 dB	9.3 dB

**Table 2:** Optimization results of roll angle (north) error.

Algorithm		Zero-Bias Stability °/*h*	Angle Random Walk °/*sqrt*(*h*)	Ramp Rate °/*h*^2^
Original signal	MAX	0.059	19.147	4.957
	MIN	0.031	10.077	2.609
WCF		0.004	1.523	0.394
Improved		8.9 dB	8.2 dB	8.2 dB

**Table 3:** Optimization results of yaw angle (sky) error.

Algorithm		Zero-Bias Stability °/*h*	Angle Random Walk °/*sqrt*(*h*)	Ramp Rate °/*h*^2^
Original signal	MAX	0.120	39.258	10.160
	MIN	0.093	30.257	7.831
WCF		0.016	5.059	1.310
Improved		7.6 dB	7.8 dB	7.7 dB

**Table 4:** Parameters of the gyro sensor.

Parameter	MEMS Gyro Sensor (*XV7011BB*)	
	MIN	MAX
Drive frequency	49.000 (*KHz*)	50.150 (*KHz*)
Scale factor stability	−2 (*%*)	+2 (*%*)
Scale change due to temperature	−3 (*%*)	+3 (*%*)
Range	−100 (°/*s*)	+100 (°/*s*)
Zero-bias stability	20 (°/*h*)	100 (°/*h*)
Angle random walk	~	~
Scale factor nonlinearity	−0.5%*FS*	+0.5%*FS*

**Table 5:** Online processing results of different algorithms.

Algorithm		Average Time Delay /*s*	Zero-Bias Stability °/*h*	Angle Random Walk °/*sqrt*(*h*)	Ramp Rate °/*h*^2^
Original signal	MAX	~	97.20	0.1800	51.470
	MIN	~	17.57	0.0490	7.740
Traditional algorithm		0.379	8.86	0.0156	4.479
WCF		0.075	2.80	0.0078	0.855
